# Synergy of multi-scale toughening and protective mechanisms at hierarchical branch-stem interfaces

**DOI:** 10.1038/srep14522

**Published:** 2015-09-29

**Authors:** Ulrich Müller, Wolfgang Gindl-Altmutter, Johannes Konnerth, Günther A. Maier, Jozef Keckes

**Affiliations:** 1 Institute of Wood Technology and Renewable Materials, University of Natural Resources and Life Science, Vienna, Austria; 2 Materials Center Leoben, Leoben, Austria; 3 Department of Materials Physics, Montanuniversität Leoben, Leoben, Austria

## Abstract

Biological materials possess a variety of artful interfaces whose size and properties are adapted to their hierarchical levels and functional requirements. Bone, nacre, and wood exhibit an impressive fracture resistance based mainly on small crystallite size, interface organic adhesives and hierarchical microstructure. Currently, little is known about mechanical concepts in macroscopic biological interfaces like the branch-stem junction with estimated 10^14^ instances on earth and sizes up to few meters. Here we demonstrate that the crack growth in the upper region of the branch-stem interface of conifer trees proceeds along a narrow predefined region of transversally loaded tracheids, denoted as *sacrificial tissue*, which fail upon critical bending moments on the branch. The specific arrangement of the tracheids allows disconnecting the overloaded branch from the stem in a controlled way by maintaining the stem integrity. The interface microstructure based on the sharply adjusted cell orientation and cell helical angle secures a zig-zag crack propagation path, mechanical interlock closing after the bending moment is removed, crack gap bridging and self-repairing by resin deposition. The multi-scale synergetic concepts allows for a controllable crack growth between stiff stem and flexible branch, as well as mechanical tree integrity, intact physiological functions and recovery after the cracking.

Internal interfaces in structured biological materials appear at all hierarchical levels and contribute decisively to the overall tissue integrity and function^1^. In the past, there has been considerable effort to understand specially the role of interfaces in the fracture resistance and deformability of biological materials at the nanoscale^2^,^3^. Impressive toughness of hard biological materials, such as bone^4^,^5^,^6^,^7^, nacre^8^,^9^ and enamel^10^,^11^ was attributed to the properties of small mineral particles^12^, soft interface organics^13^,^14^ and hierarchical microstructure^15^,^16^. These aspects are also responsible for repeated crack deflection, splitting and blunting^17^ due to the very dense interfacial network^18^, an optimized size of the hard mineral particles^12^ and stick-slip phenomena^13^. In the case of polymer-based biological materials such as wood and coir fibres, recovery of mechanical properties beyond the yield point was attributed to the interface phenomena in the cell wall mediated by crystalline cellulose and amorphous matrix of lignin and hemicellulose^19^,^20^. Up to now, functional and mechanical optimization of biological interfaces at the macro-scale has remained mainly unexplored.

In this work, we have analysed microstructural, mechanical and self-repairing mechanisms at a branch-stem interface of a Norway spruce (*Picea abies* [L.] Karst). The interface has to support not only basic physiological functions like water and nutrient transport but has to be morphologically and mechanically adapted to static and dynamic loads in order to secure the mechanical safety of the tree and a certain level of damage tolerance^21^,^22^. At the cellular level, mechanical properties of trees are primarily dependent on the magnitude of the microfibril angle (MFA), which represents the angle between the direction of the helical windings of cellulose fibrils in the secondary cell wall and the cell longitudinal axis^23^,^24^. In previous studies, it was observed that branch tissue is actually embedded in stem collars overgrowing the branch^25^ where MFA magnitude in the stem envelope was found to be adapted to the environmental and functional requirements^21^. Consequently there were no pronounced strain concentrations measured at the branch-stem cross-section during branch bending^22^. MFA distribution in the vicinity of branch-stem junction, local microstructure as well as interface mechanical behaviour have not been studied yet in detail. Since the tree possesses a complex hierarchical microstructure, the interface optimization is expected to take place at multiple length scales.

## Results

**Figure 1:**
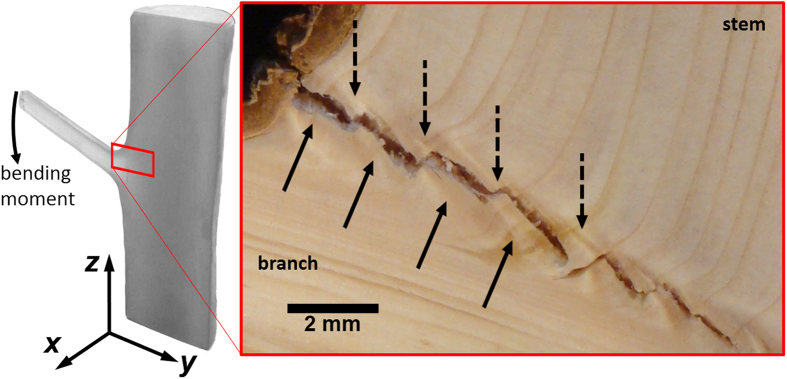
Zig-zag cracking of branch-stem interface. A radial section through the pith of the stem and branch showing the interface during crack growth initiated by the branch bending moment which results in the formation of a zig-zag crack pattern with primary and secondary cracks. The grey shaded colour of the tissue around the crack (better visible in Supplementary Fig. 1) indicates mainly the out of plane oriented tracheids and the presence of the sacrificial tissue with a relatively small fracture toughness originating from the optimized cell orientation discussed in Fig. 5.

In Fig. 1, an optical image of an exemplary tree interface during crack propagation documents the presence of a zig-zag crack pattern which was formed in the upper branch region during a bending experiment (*cf.* supplemental Video 1). The pattern consists of primary cracks which propagate along the interface parallel to the branch axis and secondary cracks which are deflected by about 90 degrees. The unique crack pattern morphology secures high energy consumption during crack growth due to multiple crack deflection (at every annual ring) which results in an increased fracture resistance. At this stage, it is not clear if this toughening mechanism originates from a tissue internal morphology or an intrinsic stress field at the interface. Moreover, after the stress relief, the zig-zag crack shape^26^,^27^ allows for a stepwise closing of the crack gap due to the elastic energy stored in the compressively stressed region of the flexible branch.

**Figure 2:**
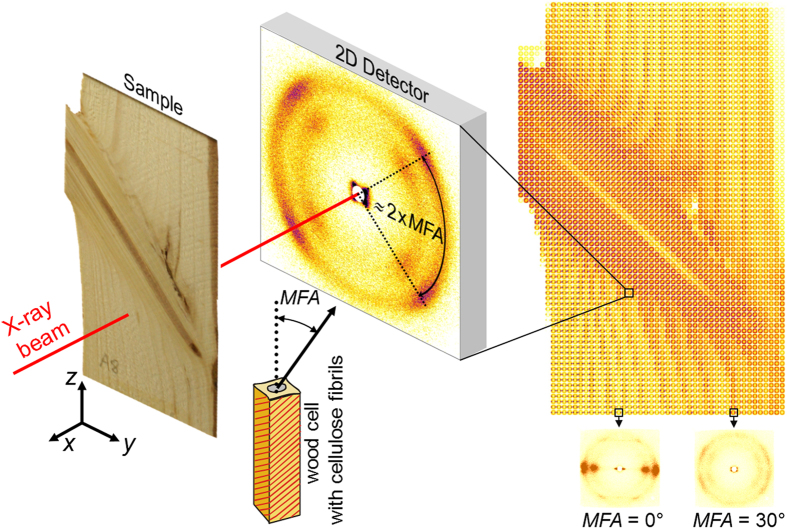
XRD analysis of the microfibril angle (MFA) distribution at the branch stem interface. A section of the interface was position-resolved analysed by moving the sample in the beam along the *y* and *z* axes. Diffraction data were collected using a 2D detector for every measurement position. The azimuthal distance between the two maxima corresponds to 2 × MFA magnitude. A composite image consists of ~3600 experimental XRD patterns collected from different sample positions and documents the variability of microstructure across the interface. 2D diffraction patterns were used to evaluate the distribution of MFA at the interface (Fig. 3), which represents the angle between the direction of the helical windings of cellulose fibrils (in red) in the secondary cell wall and the cell longitudinal axis.

**Figure 3:**
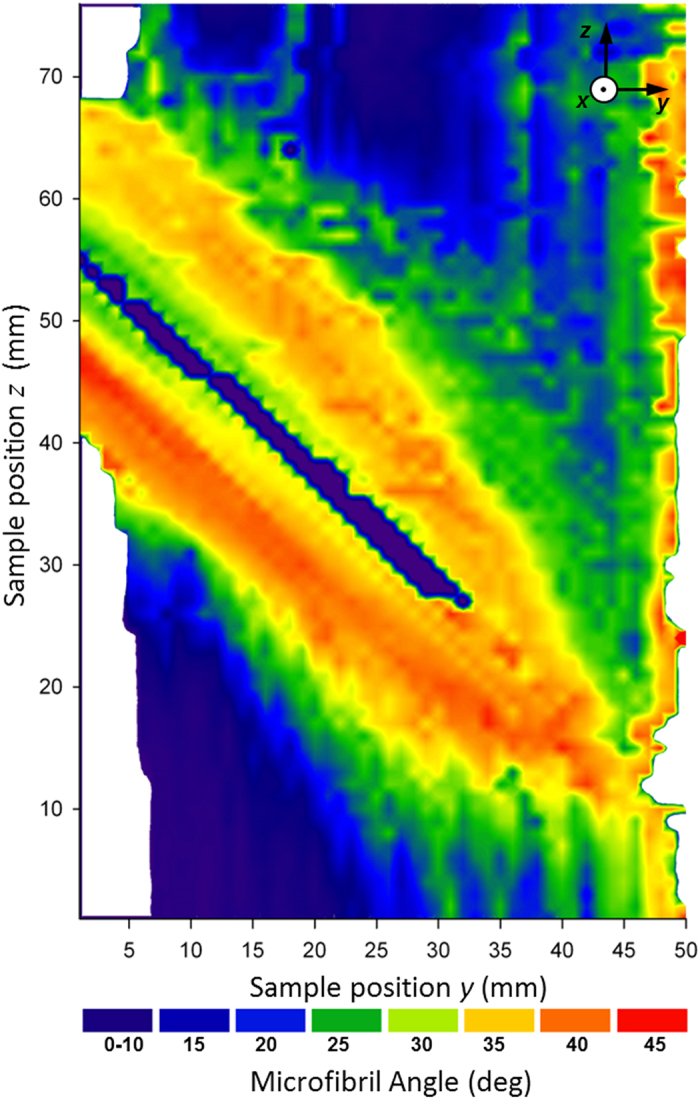
A distribution of MFA across the branch stem interface evaluated from ~3600 XRD patterns (cf. composite image in Fig. 2). Large and small MFAs in the branch and stem, respectively, indicate the presence of flexible and stiff-brittle tissues in the respective regions. The sample is presented in Supplementary Fig. 2.

A scanning X-ray diffraction (XRD) analysis of a branch-stem radial-section (Fig. 2) was used to determine MFAs distribution^23^ across the section (Fig. 3). The data indicates a relatively small MFA in the stem ranging from 10 to 20 degrees. The region of the stem bordering the interface shows medium MFAs from 25 to 30 degrees, whereas MFAs in the branch exhibit high values up to 45 degrees. Since small and large MFAs relate to stiff-brittle and ductile wood tissue responses^23^,^19^, respectively, the observed MFA variation in the secondary cell wall provides flexibility as well as toughness to the branch and stiffness as well as stability to the stem tissue. This means that during branch bending Fig. 1, the flexible branch and the surrounding tissue at the interface with the relatively high and medium MFA can be easily deformed whereas the axially loaded stem tissue above the interface with small MFA shows a comparatively stiff response.

**Figure 4:**
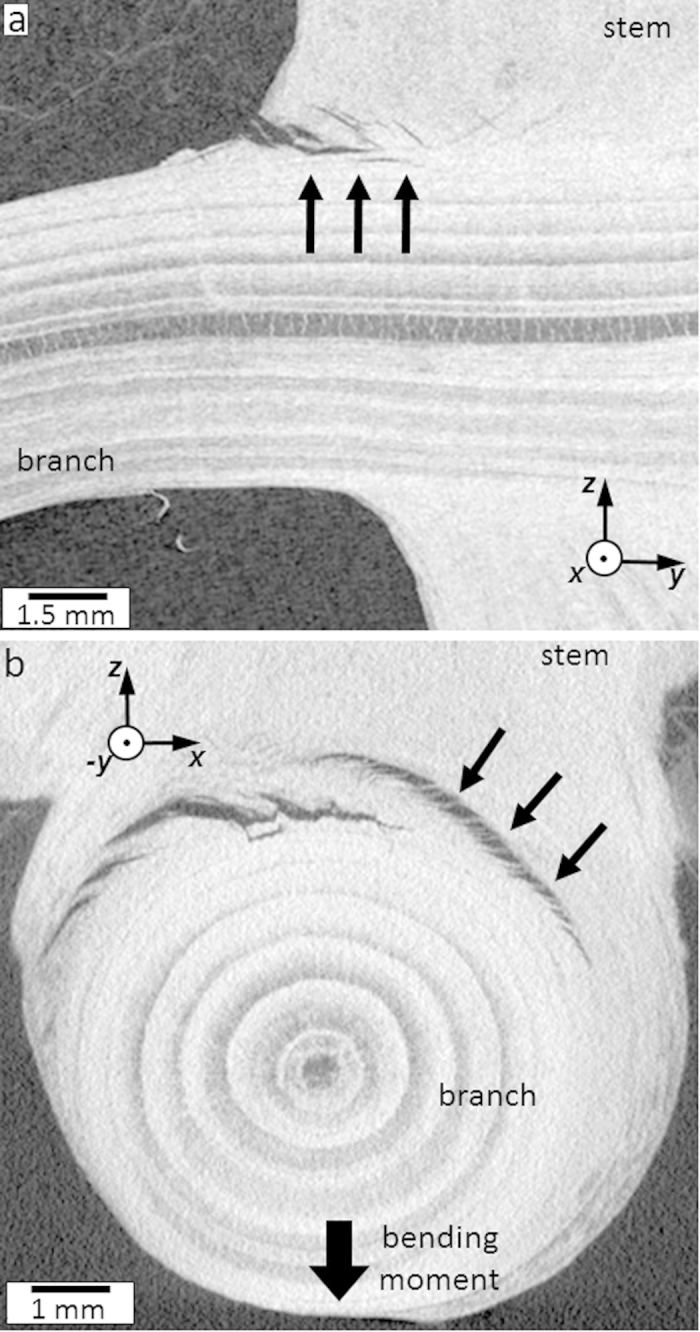
X-ray computer tomography images of a statically loaded branch-stem interface. (**a**) An early stage of the zig-zag crack pattern formation indicated by arrows is presented in the radial view. (**b**) The cracks are bridged by bundles of tracheids and wood rays of about 100 μm in thickness indicated by arrows in the axial view.

In order to analyse the morphology of the interface during crack propagation, a statically loaded branch was examined using X-ray computed tomography. Axial and tangential views document the presence of the zig-zag mechanism at early crack growth stages (Fig. 4a) as well as a crack bridging with characteristic cell bundles of about 100 μm in thickness spanning across the crack gap (Fig. 4b). The sequences of the tomography images in supplementary Videos 2 and 3 also document the presence of the crack bridges and the zig-zag crack pattern.

**Figure 5:**
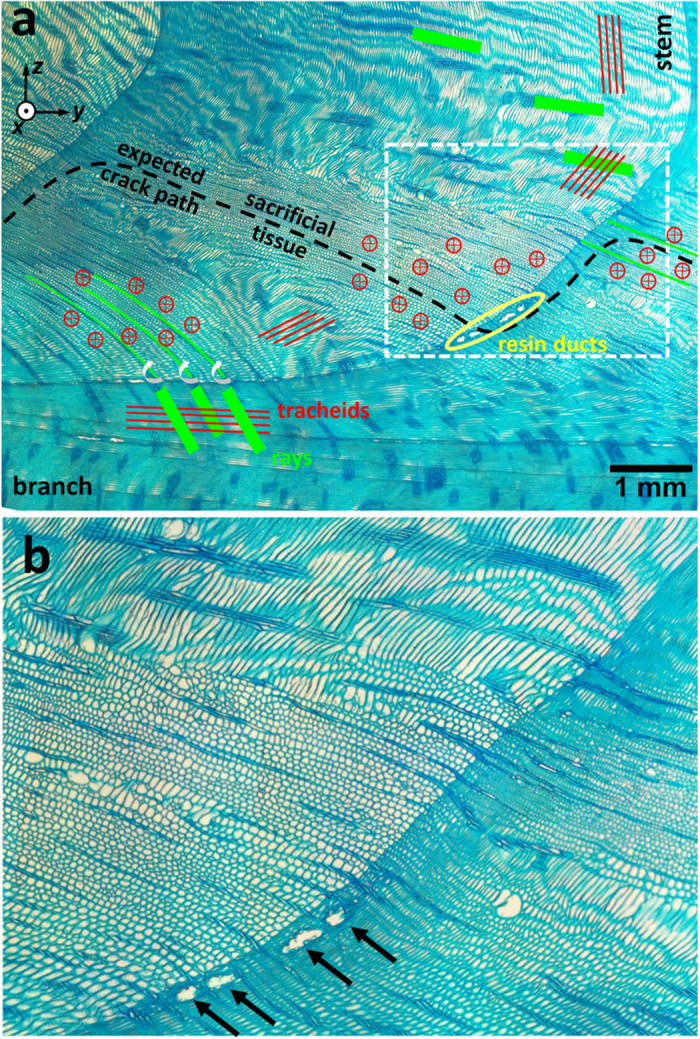
Optical micrographs of the upper region of a branch-stem interface upper region with indicated orientations of tracheid (red) and rays (green) cells. (**a**) Around the expected zig-zag crack path, there is a region of about 1 mm in thickness filled with tracheids oriented out of the image plane forming a sacrificial tissue with low fracture toughness when loaded transversally during branch bending. There is an increased density of resin ducts (marked in yellow) close to the interface which are expected to deposit resin after the fracture and fill the crack gap. In the stem and branch, the tracheids lie in the image plane. Groups of wood rays represented by dark stripes are oriented approximately perpendicular to the tracheids. The image is available in full resolution as supplementary Fig. 3. The dashed-line selection presented in (**b**) shows the tracheid orientation and the resin ducts in detail.

Origins of the specific zig-zag crack pattern (Fig. 1) and the bridging features (Fig. 4b) can be understood from the analysis of the interface microstructure presented in Fig. 5. The optical micrographs show that most of the wood tracheids (depicted as red lines in Fig. 5a) are oriented approximately parallel to the branch or the stem axes, lying in the micrographs plane of Fig. 5a. In the region of the *expected crack path*, however, the orientation of the tracheids (represented by red circles in Fig. 5a) changes in a very abrupt manner and there is a distinct narrow region with tracheids oriented perpendicular to the micrograph plane. This region denoted as *sacrificial tissue* is visible in the full resolution in the supplementary Figs 3 and 4.

**Figure 6:**
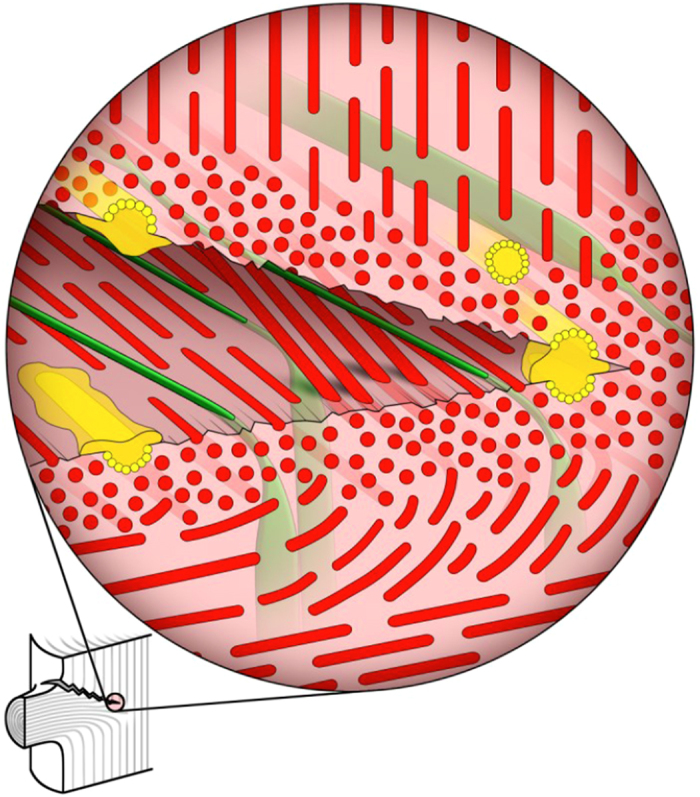
A model of the crack propagation in the sacrificial tissue at the branch-stem interface. Upon branch bending, the cracking takes place preferably in the region with transversally loaded wood cells of the sacrificial tissue represented by red dots and the crack path arrests here. Due to the finite thickness and shape of the sacrificial tissue, the crack growth is accompanied by a crack branching and repeatable crack zig-zag deflection. Critical bending moments imposed on the flexible branch with large MFA will, therefore, not be transferred on the stem cells (represented by vertical lines) but will result in disconnecting the branch from the relatively brittle stem. Wood rays (green) reinforced with tracheids (red) form tissue bundles responsible for crack bridging. The high concentration of resin ducts (yellow) activated after the cracking secures antimicrobial and hydrophobic protection.

In the case of branch bending as in Fig. 3b, the tracheids in branch upper region and in the stem are loaded axially whereas the tracheids of the sacrificial tissue are loaded transversely. Since the transverse tensile strength of wood tissue is about 10% of its axial strength^28^, the crack will propagate along the predefined crack path within the *sacrificial tissue* which is distributed step-wise along the whole interface from the pith to the bark of the stem (Fig. 5a,6). The region of the sacrificial tissue in Fig. 5a is very well defined within the annual ring structure and its distance from the branch pith increases with the formation of additional annual rings giving origin to the zig-zag pattern (cf. supplementary Fig. 4).

The crack bridging visible in Fig. 4b is mediated by wood rays and tracheids of the sacrificial tissue. The wood rays, visible as dark streaks in Fig. 5a, are oriented approximately perpendicular to the tracheids. During branch bending, the rays are loaded in shear-tension, which results in a formation of characteristic cell bundles (Fig. 4b) that are reinforced with wood tracheids (Fig. 5a).

By analysing numerous optical micrographs prepared from wood tissues of various branch-stem interface orientations before and after interface cracking, we have observed a significant concentration increase of resin ducts within the area of the sacrificial tissue (Figs 5a,b and 6). These are wood cells responsible for resin storage and delivery after potential tissue damage. It is supposed that this is an anticipatory self-repairing mechanism which is expected to support the interface mechanical interlock mechanism (Fig. 1) and serves also as an antimicrobial barrier to protect the tree against the penetration of spores and bacteria.

## Discussion

The multiple toughening and protective mechanisms discussed above are presented in a schematic model in Fig. 6. The wood tracheids within the region containing the sacrificial tissue are aligned perpendicular to the expected crack propagation direction enabling controlled cracking by loading the cells transversally. It is highly improbable that the crack would deflect into the stiff stem or flexible branch because the cells in both regions would have to crack axially. Since the crack propagation is accompanied by a tissue shear deformation, especially at the vertex of the embedded branch in the stem, bundles of tracheids reinforced with perpendicularly oriented wood rays (in green in Fig. 6) are formed in the crack gap.

The formation of the sacrificial tissue at the branch-stem interface can be explained by an adaptive feature of conifer trees in response to external displacement forces caused by branch mass^29^. The cambial activity of cells in secondary xylem is regulated by hormone signals^30^,^31^. Therefore, it is assumed that the unique stress conditions in the upper branch region during tree growth lead to intentional cell differentiation and to the formation of sacrificial tissue (Fig. 5b). At this stage, it is supposed that the formation of about a 1 mm thick region with transversally loaded cells, the sacrificial tissue region, could be induced by shear-tension stresses at the interface, which induce a very localised alternation of the wood cell orientation.

Shigo^25^ developed a macroscopic model of the fibre morphology in the vicinity of a branch-stem junction in order to explain water and nutrient transportation. The primarily physiological model of Shigo supposes that a new continuous layer of tracheids is formed every year which covers both branch as well as stem and secures water and nutrition transport. The present study deals with the mechanical optimization of the junction and shows that there are abrupt changes in the tracheidal orientation at the interface. In order to avoid an existence-threatening stem fracture, the transversally loaded cells of the sacrificial tissue disconnect the branch from the stem under a stress which is significantly smaller than the fracture stresses of the axially loaded stem cells. The mechanical model presented here does not contradict the physiological model of Shigo, but explains how the tree manages the overloading of branches. The sacrificial tissue allows disconnecting an overloaded branch from the stem in a controlled way by maintaining the stem function and structural integrity.

The concept of the zig-zag shape of the sacrificial tissue and the resulting intrinsic resistance of the interface to the crack extension is considered to be very important. Provided this morphological feature would be missing, the interface would break at relatively small bending moments on the branch and the crack would propagate directly along the interface to the stem centre. Due to the zig-zag shape of the sacrificial tissue, however, the zig-zag crack growth is accompanied by crack branching in the sacrificial tissue and crack deflection when approaching branch or stem cells. Consequently, a significantly larger crack-extension energy is needed to generate the extended crack surface^32^. Additionally, the crack-extension energy is increased because of pronounced fibre bridging of tracheids and ray cells within the sacrificial tissue. The concept of zig-zag crack behaviour was also observed in a variety of biological materials like bone^7^ and nacre^26^.

In general, the macroscopic crack pattern in Fig. 1 is a consequence of very sophisticated mechanical and structural optimization of the hierarchical interface at multiple lengths scales. On the *nanometre scale*, the adjustment of the MFAs in the stem and branch tissues guarantees strength and flexibility of both axially loaded tissues, respectively. This cell wall design hinders tissue rupture in the regions surrounding the sacrificial tissue. On the *microscopic scale*, the dedicated cell orientation causes the crack to be deflected towards the predetermined breaking zone and, upon branch bending, penetrates the tree along the predefined crack path. At the *millimetre scale*, crack bridging by shear-stressed cells again hinders the crack propagation and the bridging cell bundles can be considered also as a reinforcement of the composite formed later by the resin and the bundles themselves. At the *macroscopic scale*, the zig-zag crack path originates from the deliberate form of the sacrificial tissue spatial distribution.

An important feature of the observed toughening and protective mechanisms is their synergistic operation. Most of the mechanisms cannot exist without the other. Small MFA in the branch would result not only in its brittleness but also in the absence of the crack gap closing mechanism after the bending moment is removed. Similarly, the zig-zag shape of the sacrificial tissue allows not only for a step-wise crack growth, but it is also a prerequisite for the effectiveness of the self-repairing mechanism based on the resin deposition. A straight shape of the tissue would result in a very fast crack growth and make the protective mechanism ineffective.

We have shown that the observed mechanisms (Fig. 6) are based on the optimization of the cell wall properties, adjusted cell orientations and the distribution of the appropriate cells at the tree interface. Generally, the controlled zig-zag cracking guarantees (i) an increased energy consumption, (ii) crack propagation deceleration and (iii) increased tissue safety. We have demonstrated that nature was able to combine multi-scale concepts to optimize the mechanical behaviour of the branch-stem interface. This can be seen in contrary to the distinct mechanistic concepts identified at interfaces of lower hierarchical levels. The branch-stem interface is a masterpiece of microstructural and functional optimization and can serve as an inspiration for the design of macroscopic man-made interfaces with multi-scale mechanical concepts.

## Methods

### Preparation of branch-stem sections

Sections of freshly cut spruce trees (*Picea abies* [L.] Karst) with a diameter of 10 to 15 cm and age of 20 years were used in the experiments. Annual ring width of the juvenile wood material ranged from 0.5 to 4 mm.

Microtome sections were taken from freshly cut spruce trees by means of a conventional sledge microtome with a thickness of 15 μm. Sections were stained with methylene blue for better image contrast. A Zeiss Axioplan 2 Imaging microscope was used in transmission light mode to obtain micrographs with a magnification of 50x.

Mechanical testing for stressing the branch-stem junction was performed by using a universal testing machine (Zwick/Roell Z100, Germany). The stem body was mounted onto a carrier and the branch was continuously loaded by the movement of the cross head of the machine. The force transmission point was 100 mm from the stem surface. A force displacement curve was recorded during the experiments.

### X-ray diffraction analysis of MFA

X-ray diffraction analysis of MFA (Fig. 1b) was performed using a commercial Bruker Nanostar device applying scanning wide-angle X-ray diffraction in the transmission geometry with a beam size of 0.5 mm in diameter. The sample was moved in the X-ray beam with a step of 0.5 mm in two perpendicular directions and two dimensional diffraction patterns of 2048 × 2048 pixels recorded. The MFA magnitudes were evaluated by analysing azimuthal positions of cellulose 200 reflections.

### Computed tomography

Tomography characterization was performed using a commercial *GE phoenix nanotom* system equipped with a tungsten X-ray tube providing a spatial resolution of about 10 μm.

## Additional Information

**How to cite this article**: Müller, U. *et al.* Synergy of multi-scale toughening and protective mechanisms at hierarchical branch-stem interfaces. *Sci. Rep.* **5**, 14522; doi: 10.1038/srep14522 (2015).

## Supplementary Material

Supplementary Information

Supplementary Video 1

Supplementary Video 2

Supplementary Video 3
