# Supplementary material for: Synergy of multi-scale toughening and protective mechanisms at hierarchical branch-stem interfaces
Source: Sci Rep. 2015 Sep 29;5:14522. doi: 10.1038/srep14522 (PMC4586606; doi:10.1038/srep14522)
Supplement: Supplementary Information [file srep14522-s1.doc]

**Synergy of Multi-Scale Toughening and Protective Mechanisms at Hierarchical Branch-Stem Interface**

Ulrich Müller, Wolfgang Gindl-Altmutter, Johannes Konnerth, Günther A. Maier and Jozef Keckes

**Supplementary Figures**


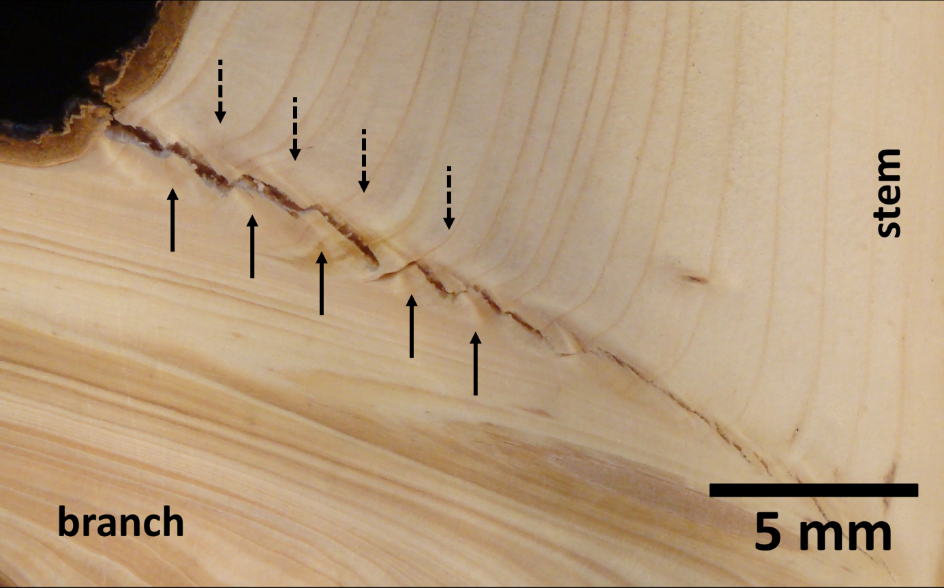


**Supplementary Figure 1**| A detail of the zig-zag crack pattern in full resolution.


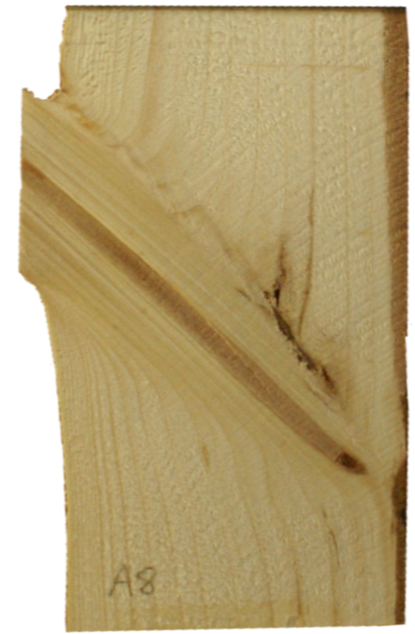


**Supplementary Figure 2**| An optical image of the sample used for the MFA characterization.


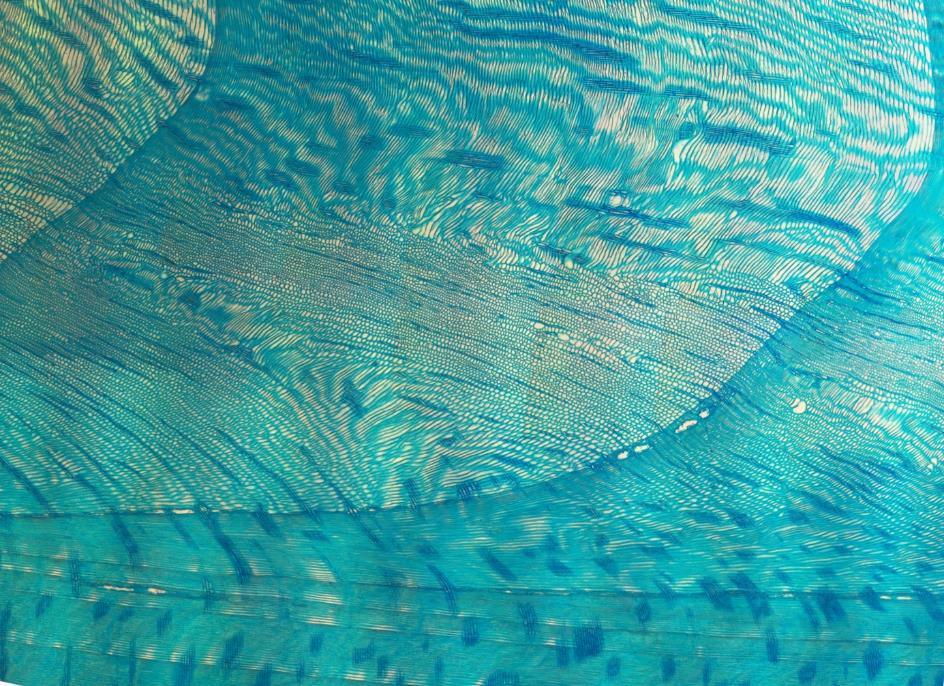


**Supplementary Figure 3**| An optical micrograph of the branch-stem interface from Fig. 5a in full resolution.


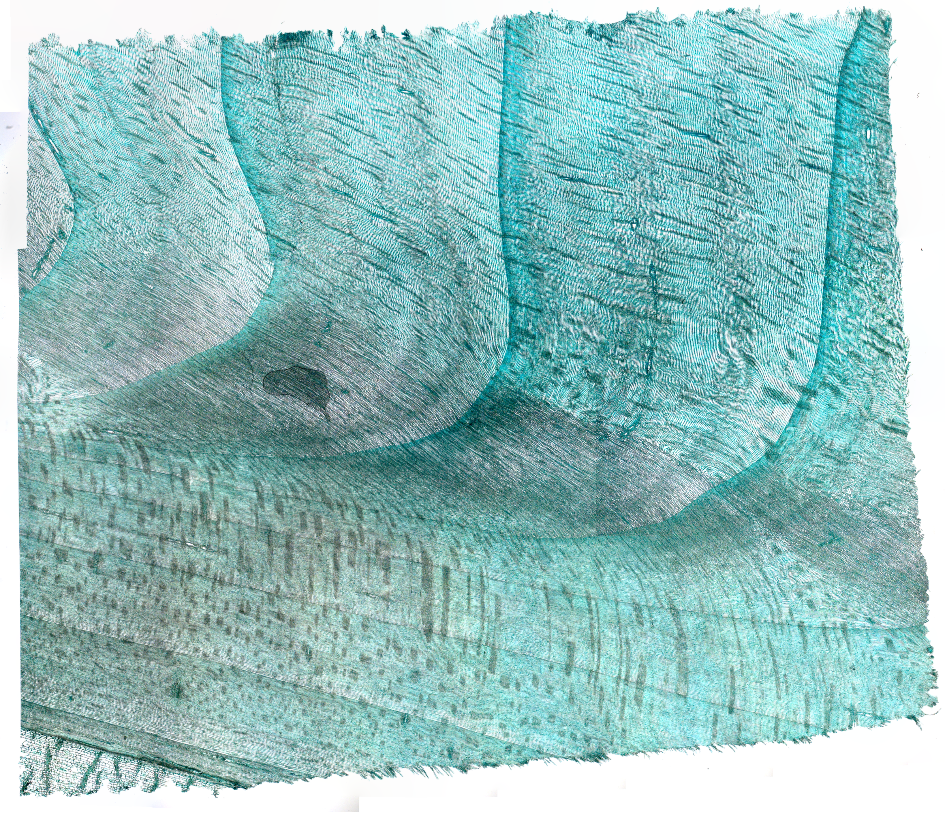


**branch**

**stem**

**Supplementary Figure 4**| An optical micrograph of the branch-stem interface in full resolution.

**Video Captions**

**Video 1** A video documenting the zig-zag cracking of the branch-stem interface during mechanical testing.

**Video 2** A sequence of computed tomography images collected from a statically loaded branch-stem interface documents the presence of cells bundles (bridging) as well as zig-zag cracking in the *radial* view.

**Video 3** A sequence of computed tomography images collected from a statically loaded branch-stem interface documents the presence of the zig-zag pattern at early stages of crack growth in the *axial* view.
